# Chronic social defeat reduces myelination in the mouse medial prefrontal cortex

**DOI:** 10.1038/srep46548

**Published:** 2017-04-18

**Authors:** Michael L. Lehmann, Thaddeus K. Weigel, Abdel G. Elkahloun, Miles Herkenham

**Affiliations:** 1 Section on Functional Neuroanatomy, Intramural Research Program, National Institute of Mental Health, NIH, Bethesda, MD 20892 USA; 2 Division of Intramural Research Programs Microarray Core Facility, National Institutes of Health, Bethesda, MD, 20892 USA

## Abstract

The medial prefrontal cortex (mPFC) plays a key role in top-down control of the brain’s stress axis, and its structure and function are particularly vulnerable to stress effects, which can lead to depression in humans and depressive-like states in animals. We tested whether chronic social defeat produces structural alterations in the mPFC in mice. We first performed a microarray analysis of mPFC gene expression changes induced by defeat, and biological pathway analysis revealed a dominant pattern of down-regulation of myelin-associated genes. Indeed, 69% of the most significantly down-regulated genes were myelin-related. The down regulation was confirmed by *in situ* hybridization histochemistry for two strongly down-regulated genes, myelin oligodendrocyte glycoprotein (*Mog*) and ermin (*Ermn*), and by immunohistochemistry for myelin basic protein. To test for stress-induced changes in myelin integrity, aurophosphate (Black Gold) myelin staining was performed on mPFC sections. Quantitative stereologic analysis showed reduced myelinated fiber length and density. Behavioral analysis confirmed that the 14-day social defeat sessions resulted in induction of depressive-like states measured in social interaction and light/dark tests. The combined data suggest that chronic social defeat induces molecular changes that reduce myelination of the prefrontal cortex, which may be an underlying basis for stress-induced depressive states.

It is well established that stress can precipitate mood and anxiety disorders and alter structure and function in key stress-related CNS areas like the hypothalamus, hippocampus, amygdala, and prefrontal neocortex^1^. Psychiatric illness-associated pathophysiological changes found in the human brain include dysregulation of the HPA axis^2^, reductions in hippocampal size^3^, and modifications in limbic frontal cortical circuitry and cellular architecture^4^. How stress alters structure and function of the nervous system is pivotal for understanding how the brain functions in health and disease.

The medial prefrontal cortex (mPFC) exerts top-down executive control of the processing of aversive and appetitive stimuli through dense interactions with subcortical regions. Abnormal function and/or structural alterations of this region is linked to the pathophysiology of many neuropsychiatric disorders, including mood disorders. Brain-imaging studies in patients with mood and anxiety disorders provide evidence of volume and connectivity reductions in cortical brain regions^5^,^6^,^7^,^8^. Such changes are hypothesized to lead to disruption of homeostatic mechanisms, resulting in destabilization and loss of synaptic connections in emotional/cognitive circuitry. This is supported by a substantial number of experimental animal studies in which lesions, pharmacological interventions, and electrophysiological and optogenetic techniques were employed to reveal the mPFC as a structure central in cognitive processes and symptoms of psychiatric disorders^9^,^10^,^11^,^12^,^13^,^14^,^15^,^16^.

Previous work from our group has demonstrated that the mPFC is key to controlling fear and emotion related behaviors and is involved in regulation of animal reaction to stressful events^16^,^17^. Here we performed a microarray-based assay of gene expression in dissected mPFC brain tissue from homecage (HC) and chronic social defeat (CSD) mice to provide a snapshot of how this structure changes after chronic psychosocial stress. Unexpectedly, we observed highly significant reductions in expression levels of genes related to myelin biogenesis and maintenance, suggesting that CSD reduces myelination of the mPFC. Stress-induced changes in the transcriptome were confirmed visually with fluorescent *in-situ* hybridization (FISH) histochemistry for two myelin-related genes, with a myelin stain, and with immunohistochemistry for myelin basic protein (MBP).

## Results

### Behavior

**Figure 1:**
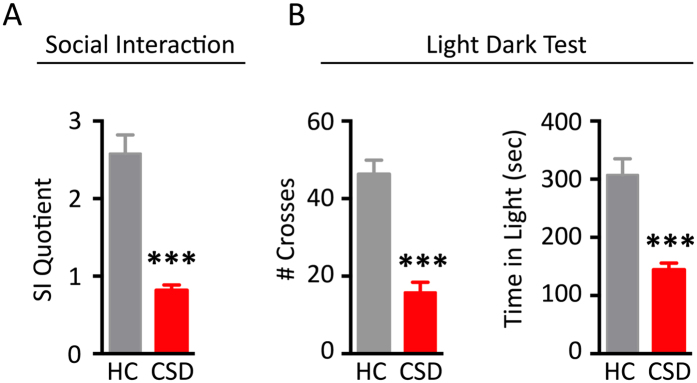
Chronic Social Defeat (CSD) significantly elevated depressive-like and anxiety-like behaviors in adult male mice. (**A**) In the social interaction task, CSD mice showed pronounced anti-social behavior measured by a significant decrease in preference for interacting with the CD-1 mouse. (**B**) In the Light/Dark test for anxiety, CSD mice showed a significant reduction in crosses between chambers, and spent more time in the dark compartment compared to HC mice. Results are expressed as mean ± SEM (*n* = 6 per group). Unpaired two-tail t-test: ***p < 0.001.

Rodents exposed to severe psychological stress show significant structural and functional alterations in the mPFC, a rodent brain structure that shares homology with structures affected in depressed patients^7^,^18^. We investigated long-term stress-induced changes in mPFC gene expression using microarray technology. To achieve this endpoint, we first used two behavioral tests to ensure that within-treatment groups were behaviorally homogeneous. 72 h prior to sample collection, we measured anxiety and sociability with the light/dark (L/D) and social interaction (SI) tests, respectively; declines in these behaviors are maladaptive responses, and they occur coincidently with anxiety-like and depressive-like behaviors measured in open field, elevated zero maze, forced swim test, and tail suspension test^16^,^17^,^19^,^20^,^21^. These two tests were used as inclusion criteria for the stressed cohort. As shown in Fig. 1, mice exposed to CSD showed significant declines in social interaction (t = 7.06, p < 0.001), and significant elevations in anxiety-like behavior that were determined by reduced crosses between light and dark compartments (t = 6.85, p < 0.001) and reduced time spent in the light portion of the L/D box (t = 5.45, p < 0.001). We also added 24 h latency between the last bout of CSD and sample collection. This allowed us to focus on long-term molecular changes induced by the CSD.

### Gene expression

**Figure 2:**
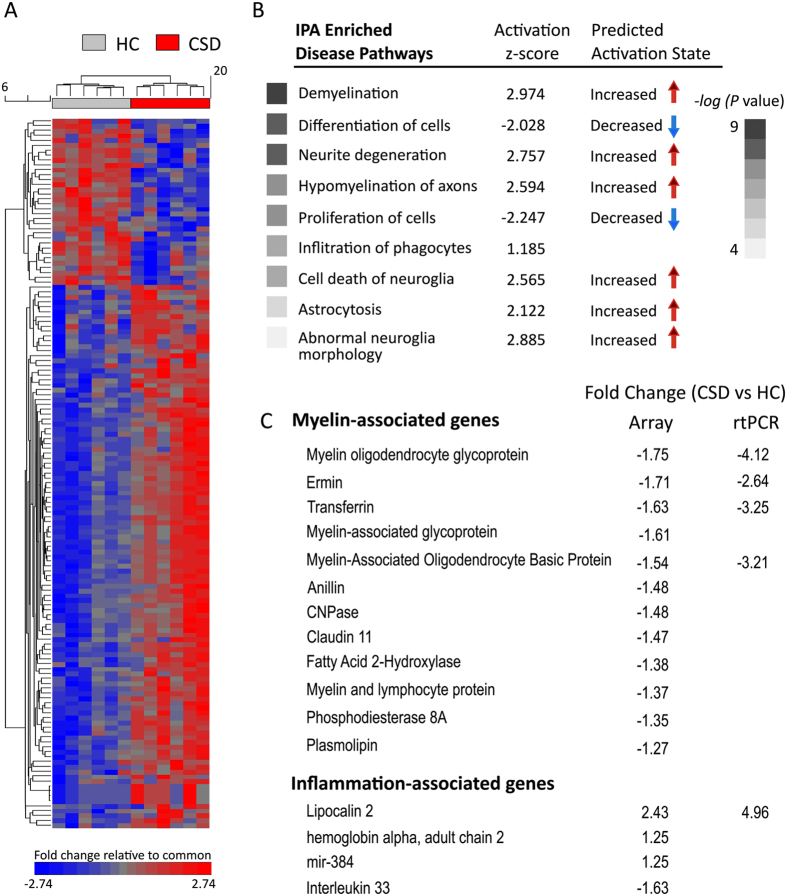
Gene expression changes induced by CSD were determined by microarray assay of the mPFC of HC vs. CSD mice. (**A**) Unsupervised hierarchical clustering and heatmap illustrates each individual’s expression pattern in 144 significant (p ≤ 0.05), differentially expressed named genes. Column dendrograms based on the gene expression data show that subjects cluster according to experimental conditions. Changes in gene expression are represented in the scale below the heatmap with red-to-blue gradient depicting an up- to down-regulation (≥2.74-fold increase → ≤ 2.74-fold decrease). (**B**) Highly enriched disease pathways was determined by Ingenuity Pathway Analysis (IPA) for all significantly altered gene transcripts. The z-score reported in the analysis describes the uniformity of the predicted activation state of the disease pathway with regard to what the change in gene expression was for the overlapping genes included in that pathway. (**C**) Selected myelin-associated genes and inflammation-associated genes are ranked by fold change. The direction and magnitude of changes were confirmed by qRT-PCR for a selected subset of up and down-regulated genes.

The raw microarray data files were deposited in the NCBI Gene Expression Omnibus at GSE84572. Of the 35,557 gene fragment expression measurements generated per sample, no sample outliers were observed. Using a fold-change threshold of 1.2, a total of 386 genes (140 named genes) differed significantly (p < 0.05) in their expression levels between CSD (*n* = 6) and HC (*n* = 6) groups at 24 h post CSD (Fig. 2A). Of the named genes, 34 were up-regulated after CSD and 108 were down-regulated in the CSD relative to HC group (Supplemental Table 1). There were 74 myelin-related genes, and all were down-regulated, thus constituting 69% of all down-regulated genes.

The top five most-upregulated genes comprised a disparate list including lipocalin 2 (*Lcn2*) (2.43-fold), musculoaponeurotic fibrosarcoma oncogene homolog G (*Mafg*) (1.35), ubiquitin specific peptidase 17-like D (*Usp17ld*) (1.31), hemoglobin alpha adult chain 2 (*Hba-a2*) (1.25), and mitochondrially encoded tRNA serine 1 (*mt-Ts1*) (1.29). The top five most-down-regulated genes were myelin oligodendrocyte glycoprotein (*Mog*) (−1.75), ermin (*Ermn*) (−1.71), transferrin (*Trf*) (−1.63), interleukin 33 (*Il33*) (−1.63), and myelin-associated glycoprotein (*Mag*) (−1.61). IPA software was used to probe relationships between genes that passed significance testing (p < 0.05), and 512 significantly enriched biological functions or disease pathways were observed. This list was greatly truncated by filtering out pathways with z-scores between the range of −1 and +1. In this context, Z-scores are used to determine whether a pathway is activated or inhibited. In an activated pathway, the actual pattern observed in the dataset matches the expected pattern in IPA resulting in a positive z-score. Using this filter, we found 19 inhibited pathways and 14 activated pathways. Examples of significantly up- or down-regulated pathways are shown in Fig. 2B. These pathways fell under three general categories—demyelination, inflammation, and cell death. The network involved with myelination appeared majorly disturbed by CSD. The function categories generated by bioinformatics analysis tools like IPA generate lists of genes that appear redundantly in multiple categories. Most genes lack dedicated and exclusive functions and therefore fall into more than one function or functional category. Thus, genes were segregated by those associated more with myelin or with inflammation. A subset of the genes is shown in Fig. 2C. The direction and magnitude of all changes were confirmed by qRT-PCR analysis for a selected subset of up- and down-regulated genes (Fig. 2C).

### Fluorescent *in situ* hybridization (FISH) for myelin oligodendrocyte glycoprotein (*Mog*) and ermin (*Ermn*) mRNA expression

**Figure 3:**
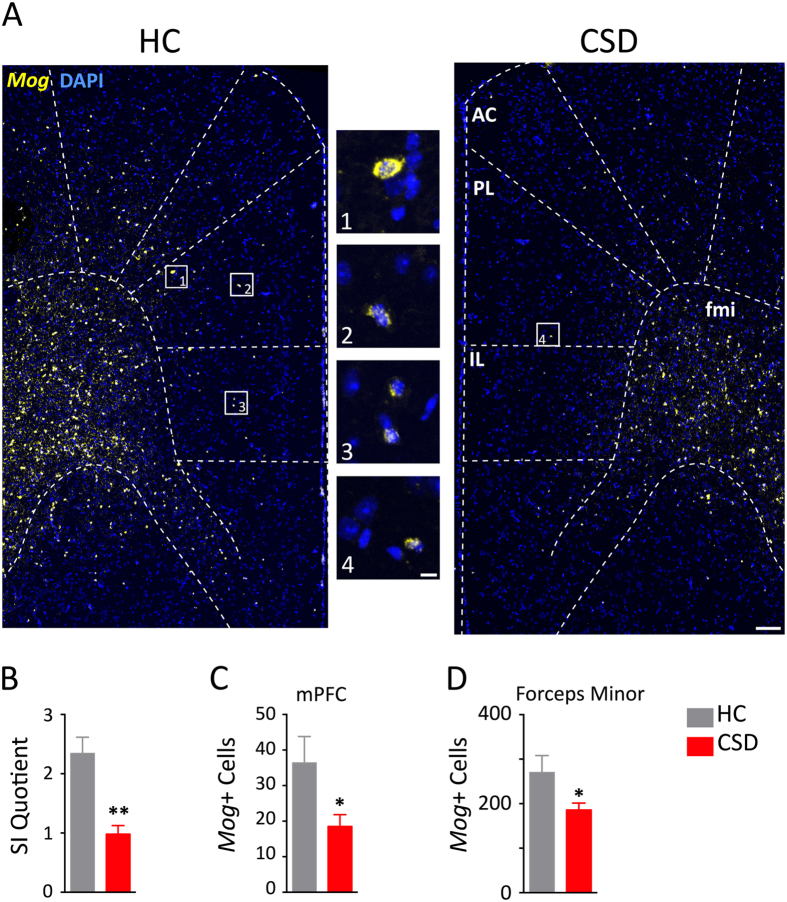
CSD reduced the expression of myelin oligodendrocyte glycoprotein (*Mog*) mRNA. (**A**) Representative fluorescent micrographs of *Mog* expression show comparative differences between HC and CSD frontal cortices. Dashed lines indicate anatomical regions and templates within which counts were made. The medial prefrontal cortex (mPFC) was defined by the anterior cingulate (AC), prelimbic (PL), and infralimbic (IL) cortices. fmi, forceps minor corpus callosum. Scale bars = 100 μm and 20 μm for low- and high-magnification photographs, respectively. (**B**) CSD mice showed markedly reduced social interaction (SI). CSD significantly reduced the number of *Mog*-positive cells in the mPFC (**C**) and fmi (**D**). Results are expressed as mean ± SEM (*n* = 5 per group). Unpaired two-tail t-test: *p < 0.05, **p < 0.005.

**Figure 4:**
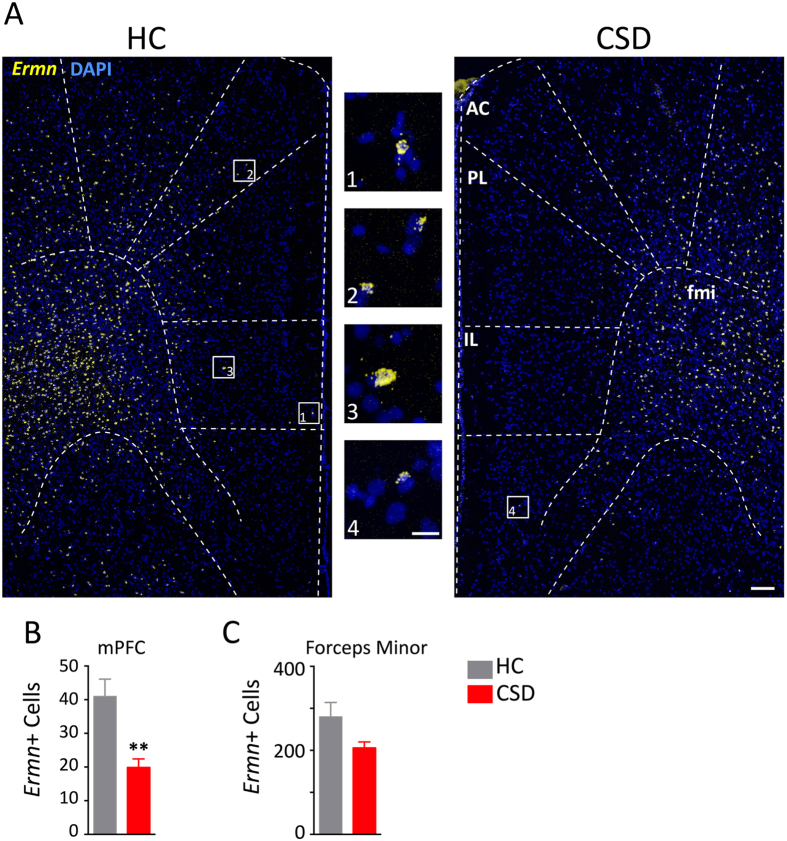
CSD reduced the expression of ermin (*Ermn*) mRNA. (**A**) Representative fluorescent micrographs of *Ermn* expression show comparative differences between HC and CSD frontal cortices. Dashed lines indicate anatomical regions and templates within which counts were made. The medial prefrontal cortex (mPFC) was defined by the anterior cingulate (AC), prelimbic (PL), and infralimbic (IL) cortices. fmi, forceps minor corpus callosum. Scale bars = 100 μm and 20 μm for low- and high-magnification photographs, respectively. (**B**) CSD significantly reduced the number of *Ermn*-positive cells in the mPFC. (**C**) Stress effects on *Ermn* expression in the fmi approached significance (*p* = 0.061). Results are expressed as mean ± SEM (*n* = 5 per group). Unpaired two-tail t-test: *p < 0.05, **p < 0.005.

We next determined whether the stress-induced reductions in myelin-related gene expression could be revealed at the histological level with cellular resolution. In a separate experiment, mice were exposed to CSD, screened for effect of stress on behavior, and then processed for myelin staining in the mPFC (Fig. 3A). CSD altered behavior in the SI task (Fig. 3B; t = 4.42, p < 0.005). FISH showed that cells expressing *Mog* mRNA were reduced in numbers throughout the gray matter and white matter of the prefrontal cortex (Fig. 3A). We counted the number of DAPI-positive cells colabeled with *Mog* probes in mPFC, which included the anterior cingulate (AC), prelimbic (PL), and infralimbic (IL) cortices, and the forceps minor (fmi). CSD significantly reduced the number of *Mog* mRNA-positive cells in the combined regions of the mPFC (Fig. 3C; t = 2.57, p < 0.04) and in the forceps minor of the corpus callosum (Fig. 3D; t = 2.64, p < 0.04). We further examined the effects of CSD on the expression of ermin, a marker of mature myelinating oligodendrocytes. CSD significantly reduced the number of *Ermn* mRNA-positive cells in the mPFC (Fig. 4A and B; t = 3.85, p < 0.002) and approached significance in the forceps minor (Fig. 4C; t = 2.02, *p* = 0.061).

**Figure 5:**
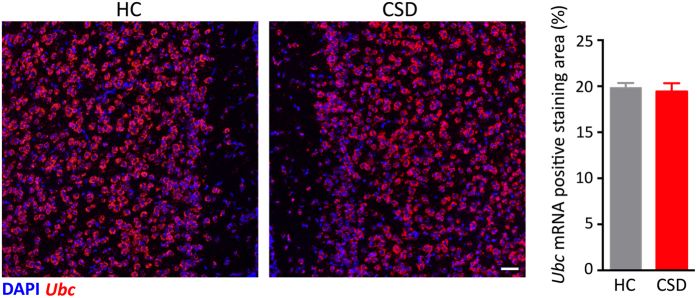
Stress does not alter the expression of ubiquitin C (*Ubc*) mRNA. (**A**) Representative fluorescent micrographs of *Ubc* expression show similarity between HC and CSD medial prefrontal cortex. Area of positive *Ubc* labeling was analyzed within this region and the graph to the right of the micrographs summarize the results. Results are expressed as mean ± SEM (*n* = 5 per group) Scale bar = 50 μm.

As an internal control, we further stained for mRNA expression of ubiquitin C (*Ubc*), a housekeeping gene expressed in all cells, that should not be altered by CSD. No difference in mPFC *Ubc* mRNA expression was observed between HC and CSD samples (Fig. 5). The data indicate both that hybridization conditions were the same in both groups and that CSD did not alter total numbers of cells in the region.

We also examined for *Mog* mRNA-positive cells in the primary motor cortex, a nonlimbic brain region, and the piriform cortex, a stress responsive region known to be activated by chronic social stress^16^,^22^. We observed a slight but non-significant decline in *Mog* mRNA-positive cells in the primary motor cortex of CSD mice (38.6 ± 2.8) vs HC mice (45.3 ± 3.9) (t = 1.39, *p* = 0.17). In the piriform cortex, stress had no effect on the expression of *Mog* mRNA-positive cells; both conditions showed similar expression levels (HC; 36.9 ± 7.6, CSD; 38.0 ± 3.1). The results suggest the effects of stress on *Mog* expression are regionally specific.

### Myelin staining of mPFC

**Figure 6:**
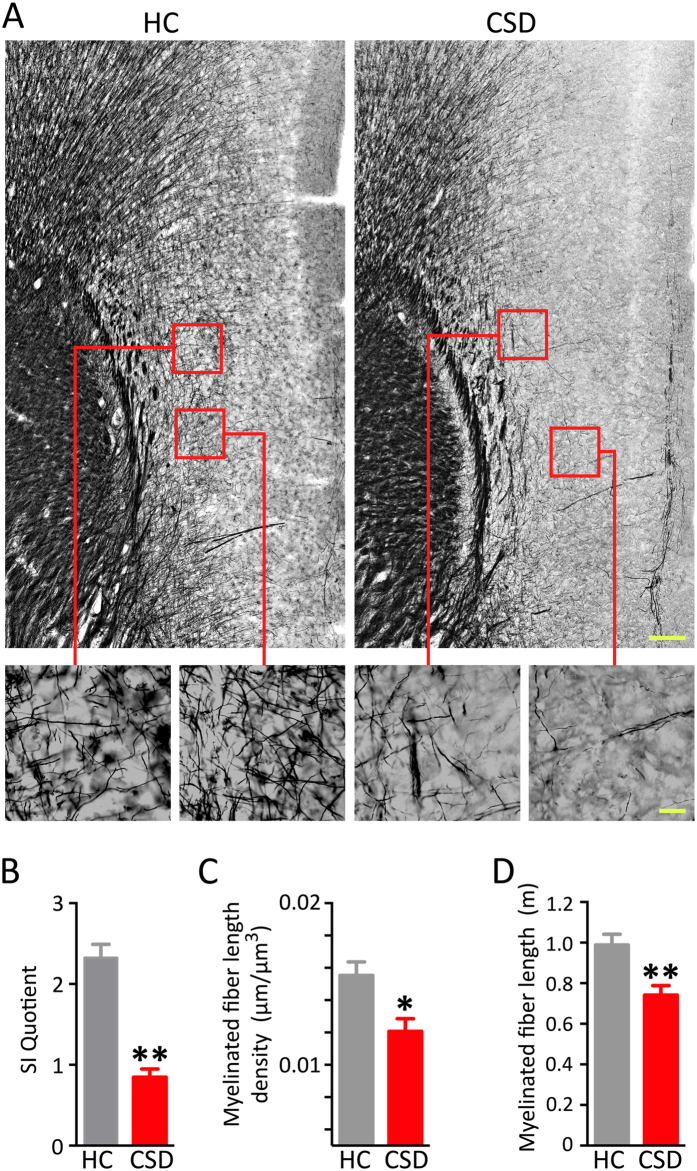
CSD decreased myelinated fiber density in the mPFC and increased social avoidance. (**A**) Representative photomicrographs of Black Gold-stained myelinated fibers show comparative differences between HC and CSD cortices. Scale bars = 100 μm and 20 μm for low- and high-magnification photographs, respectively. (**B**) CSD significantly reduced social interaction (SI). (**C**) CSD significantly reduced myelinated fiber density (**C**) and total myelinated fiber length (**D**) in the mPFC. Results are expressed as mean ± SEM (*n* = 6 per group). Unpaired two-tail t-test: *p < 0.05, **p < 0.005.

**Figure 7:**
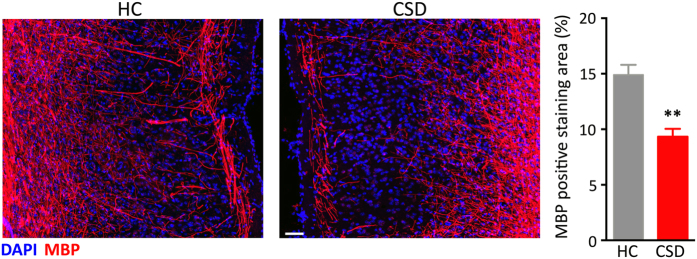
CSD reduced Myelin basic protein (MBP) immunohistochemical staining in the mPFC. Confocal images show differences between HC and CSD MBP immunoreactivity in the mPFC. CSD significantly reduced MBP immunoreactivity in the mPFC. Results are expressed as mean ± SEM (*n* = 6 per group) Scale bar = 50 μm. Unpaired two-tail t-test: **p < 0.005.

**Figure 8:**
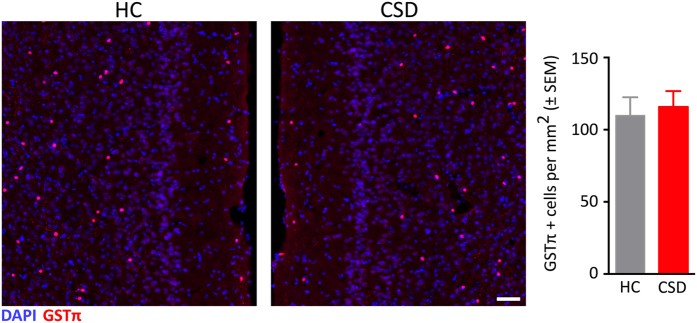
Chronic stress did not alter the number of mature oligodendrocytes (GSTπ-immunolabeled cells) in the mPFC. Confocal microscope images show similar densities of GSTπ positive cells in the mPFC of HC and CSD mice. No significant differences in GSTπ cell density were observed (graph). Results are expressed as mean ± SEM (*n* = 6 per group). Scale bar = 50 μm. Unpaired two-tail t-test (*p* > 0.05).

In a third experiment, mice were exposed to CSD, screened for effect of stress on behavior, and then processed for myelin staining, (Fig. 6A) and for MBP (Fig. 7) and GSTπ protein immunostaining (Fig. 8) in the mPFC. Mice in this experiment exposed to CSD displayed markedly lower SI behavioral scores compared to HC controls (Fig. 6B; t = 7.61, p < 0.001). Myelinated fiber length in mPFC of Black Gold-stained sections was evaluated with a stereology tool called Spaceballs that reliably measures myelin fiber length and density across a wide range of values^23^. CSD significantly reduced myelin fiber length density (MFLD) (Fig. 6C; t = 3.01, p < 0.02). Myelinated fiber length was also significantly reduced in CSD-exposed mice (Fig. 6D; t = 3.51, p < 0.005).

Reduced myelin staining in the mPFC of CSD mice was further confirmed through labeling for MBP, a major constituent of the myelin sheath in oligodendrocytes. Chronic stress significantly reduced the MBP-positive staining area in mPFC (Fig. 7; t = 4.88, p < 0.001). Stress-induced changes in myelin-related gene expression and myelin protein staining may be due to a decline in oligodendrocyte number. Therefore, we immunostained for GSTπ, a protein expressed in mature myelinating oligodendrocytes^24^,^25^,^26^. CSD however did not alter the number of oligodendrocytes in the mPFC; both groups showed a similar density of GSTπ-immunolabeled cells (Fig. 8).

## Discussion

The current study provides definitive molecular and histochemical evidence that chronic social stress reduces myelination of the mPFC in mice. The mPFC is a key structure involved with emotional regulation, and it has been associated with an inhibitory function on limbic areas activated by emotionally arousing stimuli and with a particular sensitivity to dysregulation by those stimuli^15^. Functional and structural alterations of this region have been linked to declines in behavioral affect in humans and rodent models. In the current study, transcriptional profiling of this region after CSD exposure revealed highly significant alterations in a rather small set of genes comprising the processes of neuroinflammation and myelin biology. Metaanalysis through IPA revealed that the majority of genes (69%) down-regulated by CSD were involved with myelin biosynthesis. The profound decline in expression of key myelin genes including *Mog, Mag, Mobp*, and *Ermn* is suggestive of reduced myelination. IPA pathway modeling further confirmed these classifications, and among the most significantly altered disease pathways in the IPA Analysis were demyelination, hypomyelination of axons, and cell death of neuroglia. We probed leads from the microarray suggesting stress-induced declines in myelination through two different means. First, we used fluorescent *in situ* hybridization probes to reveal the effects of CSD on the expression mRNAs for *Mog,* which codes for MOG, a glycoprotein central to the formation of the myelin sheath, and *Ermn*, which codes for ermin, a cytoplasmic protein found in the outer tongue of the myelin sheath and in the paranodal loops of mature oligodendrocytes^27^. We observed significantly reduced the number of *Mog* and *Ermn* mRNA-positive cells in the mPFC gray matter. The number of *Mog*-positive cells was also reduced in the subjacent forceps minor. Second, we observed significant declines in mPFC myelin fiber density in CSD mice using an aurophosphate stain for myelin and immunohistochemistry for MBP. Both results confirm array findings and provide compelling evidence of reduced myelination as a consequence of CSD exposure.

Several reports have used microarrays to study stress-induced changes in the rodent prefrontal cortex, but to our knowledge, this is the first to observe reductions in expression of myelin-related genes. We employed moderately stringent criteria to define differentially expressed genes, similar to other studies. However, direct gene-by-gene comparisons with these studies is problematic due to the different stress paradigms and microarray platforms used, as well as variation in brain region taken for analysis. Notably, our snapshot of transcriptional changes was captured 24 h after the last stress exposure. This delay may have removed a large subset of gene expression changes associated with acute responses to stress. We found that the vast majority of differentially expressed genes were down-regulated, with most of the up-regulated transcripts being predicted genes or pseudogenes. This pattern of gene expression is similar to other microarray studies showing mostly down regulation of genes in the PFC following chronic stress^28^,^29^,^30^. Of the few genes with elevated transcription, others also found *Lcn2* and *Hba-a2* (hemoglobin)^31^, indicating that this stress paradigm reflects findings of previous work.

*Lcn2* was the most prominent gene transcript elevated by CSD. Lipocalin-2 (LCN2) is mainly known as an antimicrobial defense mediator that scavenges a subset of bacterial siderophores, thereby restricting iron acquisition by bacteria. LCN2 is also an acute phase response protein induced by psychological stress^32^ and up-regulated in response to numerous adverse stimuli such as inflammation^33^,^34^ or cerebral ischemia^35^. LCN2 can play a role in macrophage polarization and has been shown to deactivate alveolar macrophages in pneumococcal pneumonia where it might facilitate resolution of the inflammatory response^36^. Thus, LCN2 might provide a means to prevent overwhelming inflammation and promote tissue repair and wound resolution after subsiding DAMP and PAMP signals that may occur during CSD. The exact molecular mechanisms underlying the role of LCN2 in CSD-exposed mice remains to be elucidated in future studies.

How and why stress drives hemoglobin gene expression in rodent mPFC is unknown and curious. Hemoglobin is expressed at high levels in erythrocytes, but its presence has been detected in brain in several pathological states, including chronic peripheral inflammation^37^, ischemia^38^, and social stress models^31^. Its function in brain is not well understood. Some evidence links its expression in neurons, where it plays an important role in neuronal respiration, oxidative stress, and response to injury^38^,^39^. We are currently exploring the cellular location of *hemoglobin gene* expression, and whether changes in vascular tone are responsible for the current observations.

Our main microarray finding is that over two thirds of the significantly regulated genes were myelin-related, and all of these were down-regulated by CSD. The expression patterns suggest that chronic stress decreases myelination. The prediction is striking because there is reduced myelin staining intensity in prefrontal cortical white matter of psychiatric patient brains^40^ and disruptions in microstructure of the white matter tracts connecting the frontal lobe assayed by diffusion tensor imaging (DTI) studies of human depression^41^,^42^,^43^. In addition, there is evidence for oligodendrocyte alterations across most psychiatric conditions, including autism, bipolar disorder, major depressive disorder (MDD), Alzheimer’s disease, and schizophrenia (reviewed in ref. 44). In humans, evidence for oligodendrocyte disruption has been reported using genetic, imaging, and postmortem studies (reviewed in ref. 45). Decreased oligodendrocyte-related gene expression^46^,^47^, decreased oligodendrocyte numbers and density^48^,^49^,^50^, and abnormal myelin morphology^51^ have been reported in postmortem tissue from subjects with MDD, compared to normal controls, within dorsolateral prefrontal cortex, anterior cingulate cortex, and prefrontal area BA9.

Evidence implicating decreased myelination and oligodendrocyte components associated with depressive-like states in rodents is supported in several studies showing stress-induced reduction in myelin gene expression and protein immunostaining in adolescent^52^,^53^, and adult mice^54^,^55^,^56^. Our data suggest that reduced myelination is not the result of changes in numbers of mature cells based on lack of changes in GSTπ-stained cell numbers, which is supported by a similar finding in adolescent mice^52^, whereas other studies have shown reduced numbers of new and mature oligodendrocytes in the mPFC in adult mice in chronic stress paradigms^54^,^55^.

Although it is clear that oligodendrocyte deficits are frequently observed in neuropsychiatric disorders, the actual mechanisms that induce oligodendrocyte alterations in brain remain elusive. Stress-related declines in mood are associated with dysregulation of the hypothalamic-pituitary-adrenal (HPA) axis^57^,^58^, and several reports have suggested that adrenal glucocorticoids can target oligodendrocytes directly leading to oligodendrocyte-related pathology^51^,^59^,^60^,^61^. Since CSD can produce prolonged elevations in glucocorticoids^19^, this aberrant alteration in HPA axis activity may result in altered oligodendrocyte function including declines in myelination.

Current studies have also established that in the uninjured, healthy adult brain, new myelin is continually generated in a dynamic fashion. There is evidence that changes in myelination may be modulated by experience in an activity-dependent fashion^62^,^63^,^64^. Adult mice deprived of social experiences showed declines in myelin thickness and myelin transcripts in the PFC; these changes were reversed by exposing them to socially enriched environments^65^ suggesting that myelination is a plastic response to environmental stimuli. Whether similar changes can occur with mice exposed to social defeat is unknown. However, myelination plasticity may explain the behaviorally restorative effects of enriched environments on socially defeated mice^19^,^21^.

Compelling evidence suggests that long-term changes in brain areas and circuits mediating complex cognitive and emotional behaviors represent the biological underpinnings of mood and anxiety disorders. Here we provide evidence that CSD results in reduced myelination and decline in oligodendrocyte function within the mPFC. *In vivo* magnetic resonance imaging studies have observed structural abnormalities, including white matter volume reductions, in the frontal cortex in patients with major depressive disorder^66^. Disruption of cortical-subcortical circuit integrity through microstructural changes in white matter may be involved in the etiology of major depressive disorder. Understanding biological drivers of this decline may provide new targets for therapeutic development.

## Materials and Methods

All procedures were approved by the National Institute of Mental Health Institutional Animal Care and Use Committee and conducted in accordance with the National Institutes of Health guidelines.

### Chronic social defeat (CSD)

As described previously^16^, an experimental intruder male C57BL/6 mouse was co-housed for 14 days in the home cage of a dominant aggressor CD-1 male mouse (both from The Jackson Laboratories). A perforated polycarbonate partition separated the pair. Each day, the partition was removed for 5 min to allow agonistic encounters between the pair. HC control mice were kept in group-housing conditions.

### Behavioral phenotyping

CSD and HC mice were tested in the social interaction test and light/dark test to gauge mood. One behavioral test was run per day. Automated video-based tracking of behavior was done as previously described (TopScan and TailSuspScan; Cleversys^19^,^20^). The light/dark box test was conducted on the morning prior to the 12^th^ social defeat session in a 50 × 25 × 30 cm Plexiglas box divided into dark (one-third of total area) and light compartments separated by an open door. Time spent in the light compartment and transitions between compartments during 10 min were measured. For the social interaction test, on the morning prior to the 13^th^ social defeat session, mice were placed in the open-field arena containing two upside-down wire cages. One cage contained an unfamiliar aggressor CD-1 mouse, and the other was empty. Test mice were placed in the open field and allowed to explore for 30 min. Their locations were recorded from above. Social interaction quotients following the interaction task were quantified as a ratio between duration investigating the aggressor CD-1 mouse and the empty cup.

### Microarray analysis

One day following the last social defeat, mice were rapidly anesthetized using isoflurane, perfused with 20 ml of cold saline, and decapitated. Brains were removed and placed into a brain slicer matrix (Zivic Instruments). 500 μm-thick coronal slices were collected, submerged in RNAlater (Qiagen), and incubated for 24 h at 4 °C. Under aid of a dissecting microscope, slices at levels from 1.1 mm to 2.1 mm anterior to bregma were identified. Using the forceps minor as a guide and boundary, the medial prefrontal cortex was isolated with the beveled edges of 25-ga hypodermic needles and placed in 500 μl TRIzol (Invitrogen), homogenized with a 25-ga needle and syringe, and then stored at −80 °C. Samples (*n* = 6 HC, *n* = 6 CSD) were prepared according to Affymetrix protocols. RNA quality and quantity was ensured using the Bioanalyzer (Agilent) and NanoDrop (Thermo), respectively. Total RNA (*n* = 6 HC, *n* = 6 CSD; 200 ng per sample) was prepared according to the Affymetrix protocol. The hybridization cocktail containing the fragmented and labeled cDNAs was hybridized to the Affymetrix Mouse GeneChip 1.0 ST chips. The chips were washed and stained by the Affymetrix Fluidics Station using the standard format and protocols as described by Affymetrix. The probe arrays were stained with streptavidin phycoerythrin solution (Molecular Probes, Carlsbad, CA) and enhanced by using an antibody solution containing 0.5 mg/ml of biotinylated anti-streptavidin (Vector Laboratories, Burlingame, CA). An Affymetrix Gene Chip Scanner 3000 was used in conjunction with Affymetrix GeneChip Operation Software to generate probe-level data for 29,215 mouse gene fragments per hybridized cRNA. Cel files generated by the Affymetrix AGCC program were imported into the Affymetrix Expression Consoe program and RMA (Robust Multichips Analysis) normalization was performed to generate the Chp files. The statistical programming language R was used (http://cran.r-project.org/). RMA (http://www.bioconductor.org/) was employed for probe-level data summarization and normalization. Data quality was assessed by visual inspection by Tukey box plot, covariance-based Principal Component Analysis (PCA), scatter plot, and correlation-based Heat Map. System noise was defined as the lowest observed mean data value at which the LOWESS fit of the normalized data (CV–mean) changes from non-linear to linear. Gene fragments not having at least one data value greater than system noise were discarded. Remaining data were floored to system noise and subjected to the Welch-modified t-test on a gene fragment-by-gene fragment basis with a Benjamin–Hochberg correction and false discovery rate of p < 0.05 and fold change threshold >1.20.

### Bioinformatics Analysis

Enriched biological functions for the selected gene fragments were determined using Ingenuity Pathways Analysis (IPA; Ingenuity, Inc.). Supervised analyses were conducted using separate gene fragment lists based on regulation direction. IPA is an accepted bioinformatics tool used to analyze microarray data. Of several tools available for mining array data, IPA uses a high percentage of curation by experts for multiple sources of information (see http://www.ingenuity.com/science/knowledge_base.html).

### Quantitative PCR

Quantitative RT-PCR was performed on a subset of the samples (n = 6 HC, n = 5 CSD) to validate selected candidate genes from microarray data. Total RNA was extracted using a commercial kit (Qiagen) and reverse transcribed using a Superscript III First Strand cDNA Synthesis Kit (Invitrogen). Two-step real-time RT-PCR with 2× SYBR Green Master Mix (Bio-Rad) was performed using the Bio-Rad iCycler. All primers were validated and sequenced prior to this experiment. Primer sequences are as follows: myelin oligodendrocyte glycoprotein (*Mog,* NM 010814) F: TGATTTCCCTCCCTCAACTG, R: CGTATCCTGGTTGGCAGAAT; ermin (*Ermn*, NM 029972) F: CCTTCCGCTGAGATGTCTTC, R: AGCAAAAACCCAGGAATGAA; transferrin (*Trf*, NM 133977) F: ATCCGATGCTATGACCTTGG, R: CCCTTCTTTACCACAGCCACA; myelin-associated oligodendrocytic basic protein (*Mobp*, NM 001039364) F: ACGGATGAAAACCCAGTGAG, R: CAGCAGATCCAGTCCTCCTC; lipocalin 2 (*Lcn2*, NM008491) F: CTGTCCCCTGAACTGAAGGA, R: AGGAAAGATGGAGTGGCAGA; glyceraldehyde-3-phosphate dehydrogenase (*Gapdh*, NM 008084.2) F: CAAAATGGTGAAGGTCGGTGTG, R: TGATGTTAGTGGGGTCTCGCTC. The endogenous GAPDH gene was used to normalize quantification of the mRNA target. Relative expression levels were determined by the ΔΔCt method, and the data were expressed as the fold change relative to the HC sample.

### FISH histochemistry

24 h after the end of the last defeat session, CSD and HC mice were killed by CO_2_ inhalation, and brains were quickly removed and frozen. 16 μm-thick coronal sections were placed on gelatin-coated slides and dried at 37 °C. FISH was performed according to Affymetrix ViewRNA ISH Tissue 2-Plex instructions for fresh-frozen tissue. Slides were fixed overnight at 4 °C in 10% neutral-buffered formalin (NBF). Slides were dehydrated, baked, digested with protease (all reagents from Affymetrix) for 10 min at 40 °C, and fixed with 10% NBF for 5 minutes at room temperature. Sections were incubated for 2 h at 40 °C in a hybridization oven (HybEZ, ACD) with ViewRNA Type 6 probes for mouse myelin oligodendrocyte glycoprotein (*Mog*) or Ermin (*Ermn*) transcripts. For an internal control, adjacent sections were also stained with a ViewRNA Type 1 probe for the housekeeping gene, ubiquitin C (*Ubc*). Sections were then incubated with PreAmp, Amp, and alkaline phosphatase-conjugated label probe solutions according to instructions and stained with Fast Blue for Type 6 probes and Fast Red for Type 1 probes. After counterstaining with 1:10,000 DAPI (Life Technologies) for 1 min, slides were cover-slipped with polyvinyl alcohol mounting media with DABCO (1,4-Diazabicyclo[2.2.2]octane, Sigma) to prevent fading.

Slides were imaged using Nikon Ti Eclipse fluorescent microscope and Zeiss 780 confocal microscope. Fast Blue was observed with a filter for Cy7, Fast Red was observed with a filter for Cy3. The expression levels of *Mog* and *Ermn* mRNA transcripts were quantified in the mPFC, forceps minor, the primary motor cortex (M1), and the piriform cortex (PIR). Four images per area were used for quantification. The forceps minor was defined by a 600 × 600 μm box centered within the region. The mPFC was defined by superimposing a digital template from the atlas of ref. 67 and marking the anterior cingulate, prelimbic, and infralimbic cortical boundaries. M1 was defined by a 700 × 700 μm box with the edge placed on the dorsal surface. The PIR was outlined from sections as a 200 μm-wide U-shaped band of stained cells extending lateral to medial for one-half of the width of the olfactory bulb.

Individual cells were identified on the basis of nuclear DAPI staining and were counted as expressing *Mog* or *Ermn* mRNA if one or more fluorescent dots were present in, or in close vicinity of (defined as a circle with twice the diameter of the DAPI staining) the area of DAPI staining. The analysis of *Ubc* mRNA expression was used as an internal control. The robustness of *Ubc* expression makes counting individual cells unreliable. Therefore, the area of positive *Ubc* labeling was quantitatively analyzed in a consistently positioned square (600 × 600 μm) in the mPFC containing the IL and PL using ImageJ software (http://rsb.info.nih.gov/ij/).

### Perfusions and brain sectioning

24 h following social defeat, mice were anesthetized using isoflurane and rapidly perfused transcardially with 0.9% saline followed by 4% paraformaldehyde in sodium phosphate buffer (pH 7.4 at 4 °C). Brains were rapidly removed from the skulls, postfixed overnight, and then placed in a solution containing 20% sucrose for 48 h. The frozen brains were cut into 30-μm thick coronal sections from the olfactory bulb to the end of the medulla using a freezing microtome (Leica). The slices were collected in a cold cryoprotectant solution (0.05 M sodium phosphate buffer, pH 7.3, 30% ethylene glycol, 20% glycerol) and stored at −37 °C until all samples were collected. Sections were then processed for myelin staining or immunohistochemistry.

### Black Gold myelin stain

Four brain sections spaced every 100 μm were selected between +1.8 and +1.3 mm from Bregma, containing the mPFC. Sections were washed three times for 10 min in cold KPBS then mounted onto Superfrost slides and dried overnight at room temp. Each slide contained samples from each experimental condition. All slides were stained at the same time. Slides were rehydrated in water for 2 min and then transferred into a slide mailer tube (Simport) containing 10 ml of 0.3% Black Gold II (AG105, Millipore) aurophosphate stain diluted in 0.9% NaCl preheated to 64 °C. Slides were incubated for exactly 16 min at 64 °C, then rinsed twice in RO water for 2 min each. Slides were then incubated in a preheated 1% sodium thiosulfate solution for 2 min at 64 °C, washed three times in RO water for 2 min each, dehydrated, and coverslipped with xylenes and Permount.

### Immunohistochemistry

Adjacent brain sections were stained for myelin basic protein (MBP) and glutathione S-transferase pi (GSTπ), a marker for mature oligodendrocytes. Free floating sections were washed 3x in PBS, blocked, and incubated overnight at 4 °C in rabbit anti-MBP (1:500; C-16 Santa Cruz Biotechnology) or rabbit anti GSTπ (1:300; ADI-MSA-102E, Enzo Life Sciences). Sections were rinsed and incubated for 60 min with AlexaFluor-555 anti-rabbit secondary (Invitrogen) to visualize MBP and GSTπ. DAPI was used to counterstain. Sections were mounted and analyzed using a Zeiss 780 confocal microscope. Three sections containing the prelimbic (PL) and infralimbic (IL) cortex of the mPFC from each animal were selected for quantitative analysis. Measurements were focused in a consistently positioned square (600 × 600 μm) in the mPFC containing the IL and PL. Confocal images were analyzed using ImageJ software (http://rsb.info.nih.gov/ij/).

### Stereology

Myelinated fiber length density (MFLD) was quantified in myelin-stained sections by the same observer (M.L.L.) using a computerized stereology system consisting of a Nikon E800 microscope and StereoInvestigator software (MBF Bioscience). MFLD was evaluated using the SpaceBalls probe, a 10-μm diameter sampling hemisphere for lineal features combined with a fractionator sampling scheme^68^. Each mPFC was closely delineated along the tracing window and covered an area 1.2–2.9 × 10^6^ μm^2^ depending on the location from bregma. Hemispheres were systematically and randomly placed within the stack of images through the ROI. The parameters for the grid were x (DV), 200 μm; y (ML), 100 μm; guard zone, 2 μm above and below the sphere. This yielded 40–98 frames per mPFC, ~500 frames per animal. Mean mounted section thickness was measured at every sampling location and ranged from 15.6–17.9 μm. The analysis was performed under brightfield illumination at 60x. To obtain MFLD, the total fiber length was divided by the planimetric measurement of the reference volume that was sampled, as calculated by the StereoInvestigator software.

### Statistics

All but the microarray data in this study are presented as mean ± S.E.M and analyzed with Student’s t-test. Statistical significance was set at p < 0.05.

## Additional Information

**How to cite this article**: Lehmann, M. L. *et al*. Chronic social defeat reduces myelination in the mouse medial prefrontal cortex. *Sci. Rep.* **7**, 46548; doi: 10.1038/srep46548 (2017).

**Publisher's note:** Springer Nature remains neutral with regard to jurisdictional claims in published maps and institutional affiliations.

## Supplementary Material

Supplemental Table 1
