# Supplementary material for: Chronic social defeat reduces myelination in the mouse medial prefrontal cortex
Source: Sci Rep. 2017 Apr 18;7:46548. doi: 10.1038/srep46548 (PMC5394533; doi:10.1038/srep46548)
Supplement: Supplemental Table 1 [file srep46548-s1.pdf]

Chronic social defeat reduces myelination in the mouse medial prefrontal cortex

Michael L. Lehmann*[1], Thaddeus K. Weigel[1], Abdel G. Elkahloun[2], and Miles Herkenham[1]

1. Section on Functional Neuroanatomy, Intramural Research Program, National Institute of Mental Health, NIH, Bethesda, MD 20892 USA

2. Division of Intramural Research Programs Microarray Core Facility, National Institutes of Health, Bethesda, MD, 20892 USA

*Corresponding author at: Bldg. 35, Rm. 1C911, Bethesda, MD 20892-3724. *E-mail address:* michael.lehmann@nih.gov

Supplementary Information

Supplemental Table 1.
Gene list representing all significantly up- and down-regulated named genes that met the significance testing and fold change set at ≥ 1.2. Ingenuity Pathway Analysis (IPA) Gene View function and Core Analysis was used to determine the relation of named genes to myelin.

Gene Accession	Gene Symbol	Gene Name	Related to Myelin	Ratio(CSD vs. Ctrl)	p-value(CSD vs. Ctrl)
NM_010814	Mog	myelin oligodendrocyte glycoprotein	✓	0.571419	0.000220256
NM_029972	Ermn	ermin	✓	0.583957	0.0010229
NM_001164724	Il33	interleukin 33	✓	0.613313	4.71E-05
NM_133977	Trf	transferrin	✓	0.615126	0.000254377
NM_010758	Mag	myelin-associated glycoprotein	✓	0.621342	0.00121116
NM_080450	Gjc3	gap junction protein, gamma	✓	0.63897	0.000151074
NM_001039364	Mobp	myelin-associated oligodendrocytic basic protein	✓	0.650009	0.00173292
NM_009398	Tnfaip6	tumor necrosis factor alpha induced protein 6		0.658658	0.000724577
NM_027468	Cpm	carboxypeptidase M	✓	0.661817	0.000186401
NM_028390	Anln	anillin	✓	0.673727	0.00844347
NM_001033394	Tmem88b	transmembrane protein 88B	✓	0.673731	0.000623199
NM_153520	Opalin	oligodendrocytic myelin paranodal and inner loop protein	✓	0.674506	0.00930529
NM_001146318	Cnp	2,3-cyclic nucleotide 3 phosphodiesterase	✓	0.676649	0.000730568
NM_008770	Cldn11	claudin 11	✓	0.679139	0.00184586
NM_001100182	Cyp2j12	cytochrome P450, family 2, subfamily j, polypeptide 12	✓	0.688593	0.00014991
NM_001243132	Tspan2	tetraspanin 2	✓	0.690326	0.0015753
NM_001164369	Bcas1	breast carcinoma amplified sequence 1		0.693591	0.00213572
NM_028889	Efhd1	EF hand domain containing 1		0.697746	0.000145293
NM_023113	Aspa	aspartoacylase	✓	0.700646	0.00156501
NM_001195816	Pld5	phospholipase D family, member 5	✓	0.702919	0.0391347
NM_011674	Ugt8a	UDP galactosyltransferase 8A	✓	0.712318	0.00365566
NM_010338	Gpr37	G protein-coupled receptor 37		0.718558	0.000629112
NM_144794	Tmem63a	transmembrane protein 63a	✓	0.724019	0.000128475
NM_178086	Fa2h	fatty acid 2-hydroxylase	✓	0.724908	0.0017665
NM_029726	Trdn	triadin	✓	0.727492	0.0479271
NM_053273	Ttyh2	tweety homolog 2 (Drosophila)		0.727769	0.000123491
NM_001171187	Mal	myelin and lymphocyte protein	✓	0.729054	0.0261914
NM_025961	Gatm	glycine amidinotransferase		0.732008	0.000241674
NM_019566	Rhog	ras homolog gene family, member G		0.733641	0.000172195
NM_001033302	Ciart	circadian associated repressor of transcription		0.734363	0.000947198
NM_001033349	Ankub1	ankrin repeat and ubiquitin domain containing 1		0.736999	0.00502684
NM_008803	Pde8a	phosphodiesterase 8A	✓	0.73847	0.00142099
NM_025863	Trim59	tripartite motif-containing 59	✓	0.73911	0.00979053
NM_013642	Dusp1	dual specificity phosphatase 1	✓	0.75314	0.0377119
NM_001289784	Cryab	crystallin, alpha B	✓	0.753941	0.00101801
NM_025429	Serpinb1a	serine (or cysteine) peptidase inhibitor, clade B, member 1a	✓	0.758054	0.00155456
NM_001164034	Ntf3	neurotrophin 3	✓	0.764366	0.00979126
NM_022987	Zic5	zinc finger protein of the cerebellum 5		0.769656	0.00951464
NM_001033711	Evi2a	ecotropic viral integration site 2a		0.773379	0.00115515
NM_178061	Mob3b	MOB kinase activator 3B	✓	0.776967	0.0265921
NM_021356	Gab1	growth factor receptor bound protein 2-associated protein 1	✓	0.777336	0.000576431
NM_001284392	Sept4	septin 4	✓	0.78149	0.0048467
NM_027154	Tmbim1	transmembrane BAX inhibitor motif containing 1		0.783903	0.000776133
NM_026385	Pllp	plasma membrane proteolipid	✓	0.785291	0.000279561
NM_001163182	Plekhb1	pleckstrin homology domain containing, family B	✓	0.788649	5.39E-05
NM_053190	S1pr5	sphingosine-1-phosphate receptor 5	✓	0.79066	0.00346301
NM_001290486	Lpar1	lysophosphatidic acid receptor 1	✓	0.793679	0.00453109
NM_007705	Cirbp	cold inducible RNA binding protein		0.7952	0.00371484
NM_011125	Pltp	phospholipid transfer protein	✓	0.795278	0.00111248
NM_008812	Padi2	peptidyl arginine deiminase, type II	✓	0.798479	0.00104912
NM_009801	Car2	carbonic anhydrase 2	✓	0.799423	0.00128623
NM_029789	Cers2	ceramide synthase 2	✓	0.802693	0.00395166
NM_026731	Ppp1r14a	protein phosphatase 1	✓	0.802815	0.000364817
NM_001252639	Tbc1d7	TBC1 domain family, member 7	✓	0.802849	0.000832282
NM_001166493	Rasgrp3	RAS, guanyl releasing protein 3		0.803145	0.00153148
NM_016974	Dbp	D site albumin promoter binding protein	✓	0.803532	0.000565546
NM_007421	Adssl1	adenylosuccinate synthetase like 1		0.804243	0.000133198
NM_133706	Tmem97	transmembrane protein 97	✓	0.804382	0.0122072
NM_008135	Slc6a9	solute carrier family	✓	0.806704	0.00109913
NM_016967	Olig2	oligodendrocyte transcription factor 2	✓	0.808465	0.000472958
NM_001136055	Cd82	CD82 antigen	✓	0.809488	0.000529295
NM_001025245	Mbp	myelin basic protein	✓	0.809941	0.00268091
NM_020259	Hhip	Hedgehog-interacting protein	✓	0.809996	0.00940069
NM_008161	Gpx3	glutathione peroxidase 3	✓	0.810075	0.00710155
NM_009894	Cideb	cell death-inducing DFFA-like effector b		0.810583	0.000140875
NM_001033481	Myrf	myelin regulatory factor	✓	0.811717	0.00184496
NM_007657	Cd9	CD9 antigen	✓	0.81296	0.00420797
NM_010255	Gamt	guanidinoacetate methyltransferase	✓	0.813023	0.000643152
NM_145148	Frmd4b	FERM domain containing 4B	✓	0.813158	0.000604498
NM_001161767	Galnt6	UDP-N-acetyl-alpha-D-galactosamine	✓	0.814243	0.000810067
NR_002843	Rnu3b4	U3B small nuclear RNA 4		0.814252	0.00666752
NM_001285927	Dock10	dedicator of cytokinesis 10	✓	0.814589	0.038941
NM_013807	Plk3	polo-like kinase 3		0.81521	0.00498146
NM_001242349	Ano1	anoctamin 1	✓	0.815236	0.0261476
NM_001161847	Sgk1	serum/glucocorticoid regulated kinase 1		0.817193	0.0348447
NM_001005868	Erbb2ip	Erbb2 interacting protein	✓	0.818024	0.00650577
NM_016718	Ninj2	ninjurin	✓	0.819027	6.88E-05
NR_001584	Speer8-ps1	spermatogenesis associated glutamate (E)-rich protein 8		0.81978	0.00745951
NM_029726	Trdn	triadin		0.820511	0.0119645
NM_019821	Gltp	glycolipid transfer protein	✓	0.820772	2.25E-05
NM_001039558	Smco3	single-pass membrane protein with coiled-coil domains		0.820786	0.00269955
NM_001290561	Plp1	proteolipid protein (myelin) 1	✓	0.820859	0.00192107
NM_145434	Nr1d1	nuclear receptor subfamily 1, group D, member 1	✓	0.821246	0.00190191
NM_080708	Bmp2k	BMP2 inducible kinase	✓	0.821543	0.00255246
NM_007646	Cd38	CD38 antigen	✓	0.823	0.0291953
NM_024236	Qdpr	quinoid dihydropteridine reductase	✓	0.823212	0.00131708
NM_023543	Chn2	chimerin 2	✓	0.823394	0.00421343
NM_001048227	Dbndd2	dysbindin	✓	0.823413	0.00119952
NM_001104623	Vmn2r12	vomeronasal 2, receptor 12		0.823738	0.0269375
NM_018881	Fmo2	flavin containing monooxygenase 2		0.823944	0.0408503
NM_026481	Tppp3	tubulin polymerization-promoting protein family member 3		0.824684	0.00098441
XM_006520082	Spef2	sperm flagellar 2	✓	0.824752	0.00911821
NM_011932	Dapp1	dual adaptor for phosphotyrosine and 3-phosphoinositides 1		0.826104	0.00672628
NM_177390	Myo1d	myosin ID	✓	0.826338	0.00429985
NM_001037736	Arhgef10	Rho guanine nucleotide exchange factor (GEF) 10	✓	0.826378	0.00822896
AB062122	Prkcq	protein kinase C, theta	✓	0.826519	0.00117898
XM_006516236	Foxn3	forkhead box N3		0.827274	0.0017063
NM_013467	Aldh1a1	aldehyde dehydrogenase family 1, subfamily A1	✓	0.827392	0.0421428
NM_178061	Mob3b	MOB kinase activator 3B		0.827936	0.000225582
NM_001024720	Hmcn1	hemicentin 1		0.827977	0.00592316
NM_007494	Ass1	argininosuccinate synthetase 1	✓	0.82927	0.0014316
ENSMUST00000058538	Rassf8	Ras association (RalGDS/AF-6) domain family (N-terminal		0.829297	0.00869874
ENSMUST00000083294	Gm25789			0.829457	0.0325639
ENSMUST00000141777	9030624G23Rik			0.830467	0.00466761
NM_013831	Pstpip2	proline-serine-threonine phosphatase-interacting protein 2		0.830468	0.0214751
NM_019980	Litaf	LPS-induced TN factor		0.830808	0.00320293
XM_006509444	Enpp6	ectonucleotide pyrophosphatase/phosphodiesterase 6	✓	0.830976	0.0139285
NM_139269	Pla2g16	phospholipase A2, group XVI	✓	0.831887	0.00220595
NR_004439	Rprl2	ribonuclease P RNA-like		1.20237	0.00557677
ENSMUST00000103688	Traj57	T cell receptor alpha joining 57		1.20554	0.0137922
ENSMUST00000083912	Gm25937			1.20669	0.000669621
ENSMUST00000082624	Gm24620			1.20828	0.00265165
ENSMUST00000158343	Gm22113			1.21236	0.0338156
NM_001113734	Mageb16	melanoma antigen family B, 16		1.21391	0.00337046
ENSMUST00000082721	Gm25788			1.21573	0.00227843
AK038707	9330155M09Rik			1.21862	0.0256534
NR_028576	Scarna13	small Cajal body-specific RNA		1.22048	0.024615
ENSMUST00000093430	Gm7125			1.22076	0.00713747
NR_033537	Gm10754			1.22366	0.0394823
NM_001081448	Vmn2r84	vomeronasal 2, receptor 84		1.22378	0.0230501
NM_001105152	Vmn2r48	vomeronasal 2, receptor 48		1.22384	0.0243285
XR_399496	Gm10855			1.22471	0.0366363
ENSMUST00000180343	Gm25947			1.22566	0.000994294
ENSMUST00000082775	Gm22509			1.22712	0.0100717
ENSMUST00000076071	Gm10115			1.23567	0.0329638
ENSMUST00000179471	Gm22951			1.23778	0.00374321
NR_028513	Snora73b	small nucleolar RNA, H/ACA box 73b		1.23879	0.0144302
AK034486	Gm16495			1.23954	0.0267658
ENSMUST00000119037	Gm12986			1.25005	1.19E-05
NM_001083955	Hba-a2	hemoglobin alpha, adult chain 2		1.25131	0.0450926
NR_029910	Mir384	microRNA 384		1.25318	0.0177876
ENSMUST00000082975	Gm25401			1.25324	0.00270533
ENSMUST00000082473	Gm23926			1.26506	0.00476164
ENSMUST00000118690	Gm12901			1.26619	0.00103745
ENSMUST00000179845	Gm25870			1.27419	0.0053011
ENSMUST00000082403	mt-Ts1	mitochondrially encoded tRNA serine 1		1.28148	0.00429623
ENSMUST00000178527	Gm25646			1.29211	0.013415
NM_001001559	Usp17ld	ubiquitin specific peptidase 17-like D		1.3037	0.03187
NR_024201	Rnu7	U7 small nuclear RNA		1.33685	0.00791275
ENSMUST00000058162	Mafg	v-maf musculoaponeurotic fibrosarcoma oncogene family		1.3591	0.0143059
ENSMUST00000082598	Gm23614			1.45519	0.000734071
NM_008491	Lcn2	lipocalin 2		2.43547	0.0220145
